# Evidence for Critical Role of Lymphocyte Cytosolic Protein 1 in Oral Cancer

**DOI:** 10.1038/srep43379

**Published:** 2017-02-23

**Authors:** Nao Koide, Atsushi Kasamatsu, Yosuke Endo-Sakamoto, Sho Ishida, Toshihiro Shimizu, Yasushi Kimura, Isao Miyamoto, Shusaku Yoshimura, Masashi Shiiba, Hideki Tanzawa, Katsuhiro Uzawa

**Affiliations:** 1 Department of Oral Science, Graduate School of Medicine, Chiba University, Chiba, Japan; 2 Department of Dentistry and Oral-Maxillofacial Surgery, Chiba University Hospital, Chiba, Japan; 3 Department of Oral Surgery, Kashima Rosai Hospital, Ibaraki, Japan; 4 Department of Oral and maxillofacial Surgery Research Institute, National Defense Medical College, Saitama, Japan; 5 Department of Dentistry and Oral-Maxillofacial Surgery, Japanese Red Cross Fukaya Hospital, Saitama, Japan; 6 Department of Clinical Oncology, Graduate School of Medicine, Chiba University, Chiba, Japan

## Abstract

Lymphocyte cytosolic protein 1 (LCP1), a member of actin-binding protein of the plastin family, has been identified in several malignant tumors of non-hematopoietic sites, such as the colon, prostate, and breast. However, little is known about the roles of LCP1 in oral squamous cell carcinomas (OSCCs). This present study sought to clarify the clinical relevance of LCP1 in OSCCs and investigate possible clinical applications for treating OSCCs by regulating LCP1 expression. We found up-regulation of LCP1in OSCCs compared with normal counterparts using real-time quantitative reverse transcription polymerase chain reaction (qRT-PCR), immunoblotting, and immunohistochemistry (*P* < 0.05). We used shRNA models for LCP1 (shLCP1) and enoxacin (ENX), a fluoroquinolone antibiotic drug, as a regulator of LCP1 expression. In addition to the LCP1 knockdown experiments in which shLCP1 cells showed several depressed functions, including cellular proliferation, invasiveness, and migratory activities, ENX-treated cells also had attenuated functions. Consistent with our hypothesis from our *in vitro* data, LCP1-positive OSCC samples were correlated closely with the primary tumoral size and regional lymph node metastasis. These results suggested that LCP1 is a useful biomarker for determining progression of OSCCs and that ENX might be a new therapeutic agent for treating OSCCs by controlling LCP1 expression.

The plastin family, which is comprised of actin-binding proteins, is conserved evolutionary and expressed in such as yeast, plant, and animal cells^1^. Three isoforms of plastin (T-, I-, and L-types) have been identified in mammals. Among them, L-plastin, lymphocyte cytosolic protein 1 (LCP1), is expressed in hematopoietic cellular lineages and many types of cancers^1^. While many kinds of the actin-binding proteins modulate dynamics of the actin cytoskeleton, recent studies have concerned LCP1 in regulation of actin dynamics^2^. Activated LCP1 induced high cellular adhesion and increased actin binding and actin assembly^2^.

LCP1 is found in many kinds of tumoral cells of non-hematopoietic origin, such as in the colon, prostate, and breast. LCP1 expression is correlated positively with advanced tumoral stages and severity in colon and breast cancers and is assumed a potential prognostic indicator^3^,^4^. LCP1 is overexpressed and play an important role in tumor cell functions in colon cancer, furthermore, LCP1 gene serve as gender- and/or stage-specific molecular predictors of tumor recurrence as well as potential therapeutic targets^5^. Similar to those cancers, LCP1 is participated in tumoral invasion and metastasis in prostate cancer cells, and its knockdown experiment is potentially a useful approach for treating tumors^1^,^6^. In prostatic epithelial cells, the expression of LCP1 is associated with the malignant status and is regulated by steroid hormone receptors^7^. Moreover, an oral broad-spectrum fluoroquinolone, enoxacin (ENX), controlled expression of LCP1 and led to similar phenotypes of LCP1 knockdown cells in prostate cancer^8^. In addition, cellular invasiveness of malignant melanoma cells requires not only LCP1 expression status but also the phosphorylation levels of LCP1^9^. However, the functional significance of LCP1 expression in oral squamous cell carcinomas (OSCCs) for tumoral cellular proliferation and metastasis remains uncertain.

In the present study, we sought to clarify the clinical relevance of LCP1 in OSCCs and valuate a new candidate for medical treatment of OSCCs by drug repositioning of an antibiotic agent, enoxacin.

## Results

### Up-Regulation of LCP1 in OSCC Cell Lines

**Figure 1:**
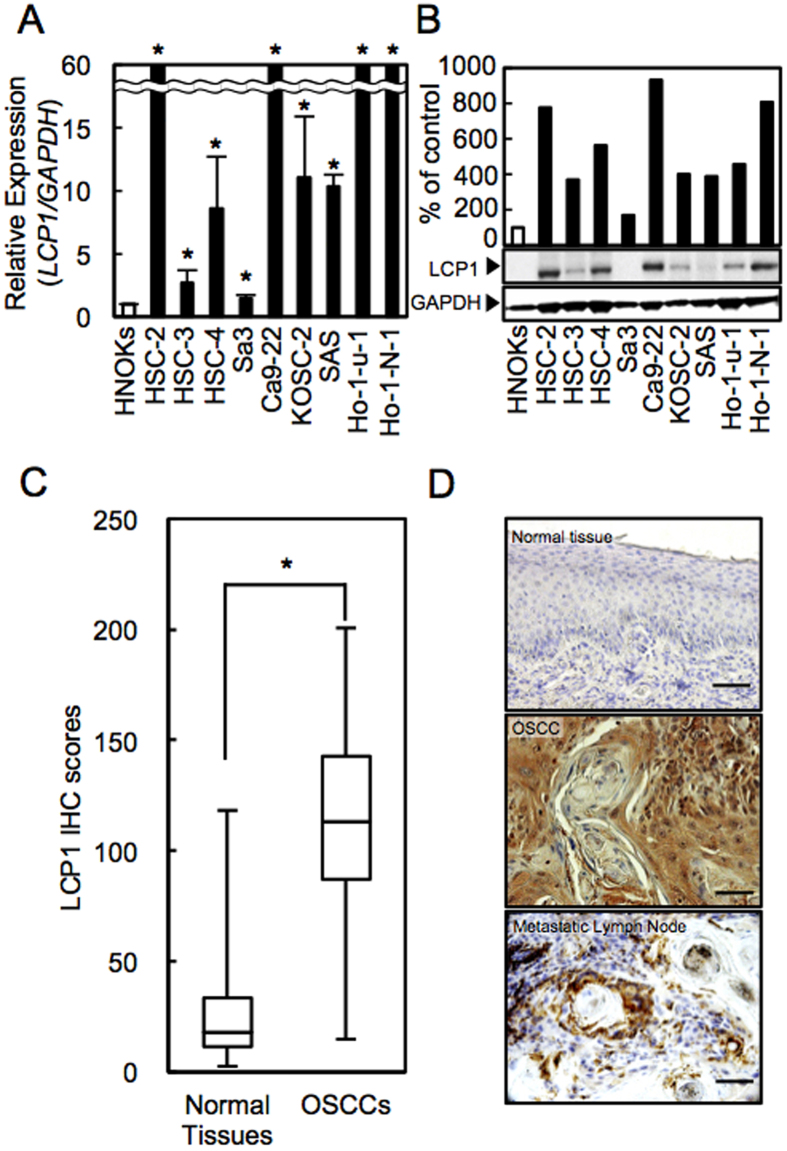
LCP1 expression in OSCC-derived cell lines and in primary OSCCs. **(A)** Quantification of *LCP1* mRNA expression in OSCC-derived cell lines by qRT-PCR analysis. **(B)** Representative immunoblot analysis of LCP1 protein expression. Densitometric LCP1 protein data are normalized to GAPDH protein levels. The values are expressed as a percentage of the HNOKs. **(C)** The LCP1 IHC scores of normal oral tissues and OSCCs. **(D)** Representative IHC results for LCP1 protein in normal tissue, primary OSCCs, and metastatic regional lymph nodes. Original magnification, x 400. Scale bars, 50 μm.

To evaluate the status of LCP1 expression as a cancer-related gene, we conducted real-time quantitative reverse transcription polymerase chain reaction (qRT-PCR) and immunoblot analyses with nine OSCC-derived cell lines and human normal oral keratinocytes (HNOKs). *LCP1* mRNA expression was significantly up-regulated in all OSCC-derived cell lines compared with the HNOKs (Fig. 1A, *P* < 0.05). Figure 1B gives representative results of immunoblot analysis. The LCP1 protein also increased in all OSCC cell lines compared with the counterpart.

### Evaluation of LCP1 Status in Primary OSCCs

We evaluated the LCP1 expression in primary OSCCs by immunohistochemistry (IHC) and the IHC scoring system^10^. The IHC scores of LCP1 in oral normal tissues and primary OSCCs ranged from 2.7 to 118.2 (median, 18.2) and 14.9 to 200.7 (median, 112.9). These IHC scores in primary OSCCs were significantly greater than in normal oral tissues (Fig. 1C, *P* < 0.05). Representative IHC figures for LCP1 protein in normal tissues, primary OSCCs, and metastatic lymph node were shown in Fig. 1D. Intense LCP1 immunoreactivity was observed in primary OSCCs and metastatic lymph nodes, whereas the normal oral tissues showed almost negative immunostaining.

### Establishment of LCP1 Knockdown Cells

**Figure 2:**
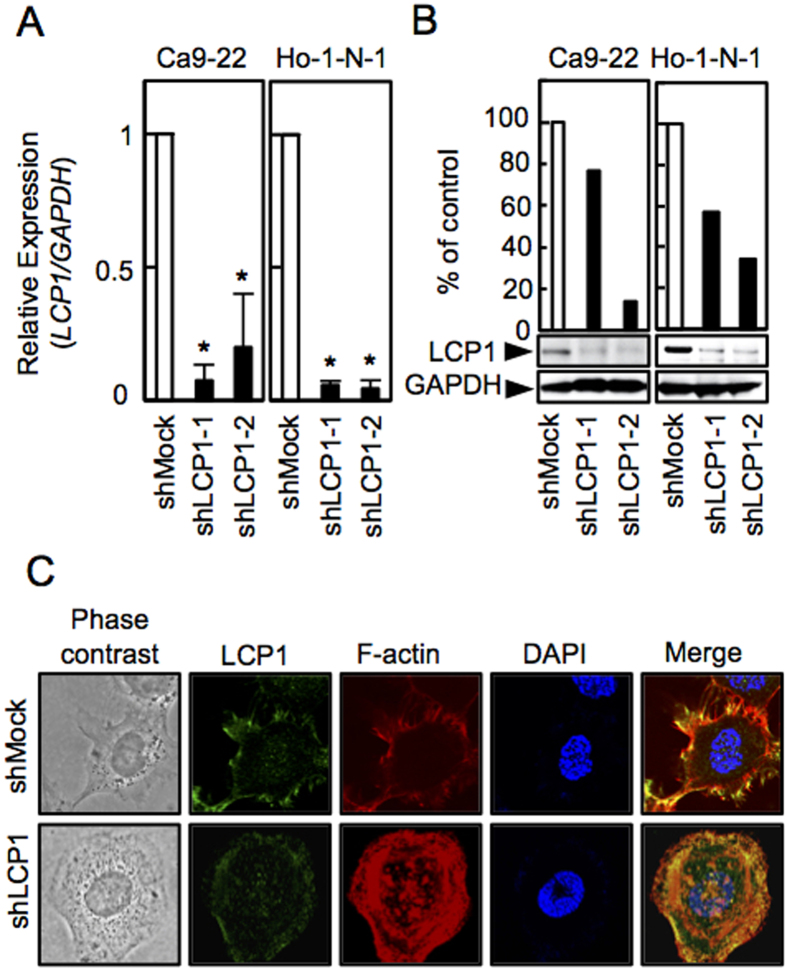
Establishment of LCP1 knockdown cells. **(A)** Expression of *LCP1* mRNA in shMock and shLCP1 cells (Ca9-22 and Ho-1-N-1-derived transfectants). **(B)** Immunoblot analysis of the LCP1 protein levels in shLCP1 cells and shMock cells. **(C)** Immunofluorescence of LCP1 and F-actin in shLCP1 cells and ahMock cells.

Because overexpression of LCP1 was frequently seen in OSCC *in vitro* and *in vivo* (Fig. 1), we transfected LCP1 shRNA or shMock vectors into OSCC cells (Ca9-22, Ho-1-N-1). To investigate the efficiency of the transfection, we conducted qRT-PCR and immunoblot analyses. The *LCP1* mRNA expression levels in the shLCP1 cells was lower than in the shMock cells (Fig. 2A, *P* < 0.05). Similarly, the LCP1 protein level in the shLCP1 cells decreased compared with the counterparts (Fig. 2B). To clarify the effect of LCP1 knockdown on localization of F-actin, we performed immunofluorescence (IF), which showed that LCP1 and F-actin were co-localized in the cytosol near the plasma membrane in shMock cells, whereas LCP1 and F-actin were expressed throughout the cytosol in shLCP1 cells (Fig. 2C). Additionally, in our clinical samples, the cancer cells at the invasive front revealed strong immunoreaction for both LCP-1 and F-actin. This suggest that these molecules may collaborate for the tumor invasion. These results have indicated in the Supplemental Fig. S1.

### Functional Assays

**Figure 3:**
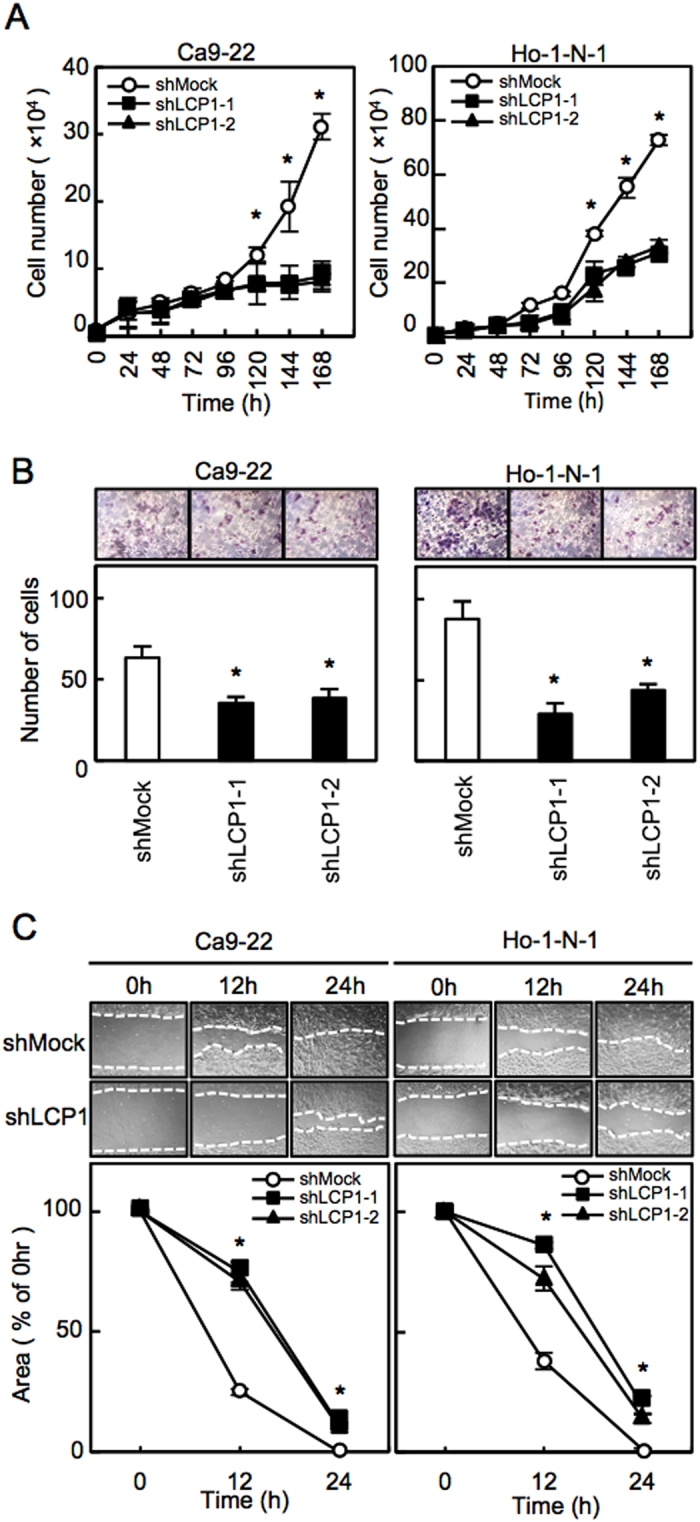
Functional assays of LCP1 knockdown cells. **(A)** Proliferation assays of shMock cells and shLCP1 cells. The results are expressed as the mean ± standard error of the mean of the values from three assays. **(B)** Invasion assay of shMock cells and shLCP1 cells. The mean value is calculated from data obtained from three separate chambers. **(C)** Migration assay of shMock cells and shLCP1 cells. The mean value is calculated from data obtained from three separate chambers.

A proliferation assay was performed to evaluate the effect of LCP1 knockdown on cell growth showed that the cell growth of shLCP1 cells was significantly inhibited compared with shMock cells after 120 h (Fig. 3A, *P* < 0.05; Student’s t-test). We also performed invasion and migration assays to evaluate the effect of LCP1 knockdown on cell invasiveness and migratory abilities. The number of invading shLCP1 cells significantly decreased compared with shMock cells after 48 h (Fig. 3B, *P* < 0.05; Student’s t-test), and the wound size significantly decreased in shMock cells after 12 h, whereas in the shLCP1 cells (Fig. 3C, *P* < 0.05; Student’s t-test).

### Enoxacin Treatment

**Figure 4:**
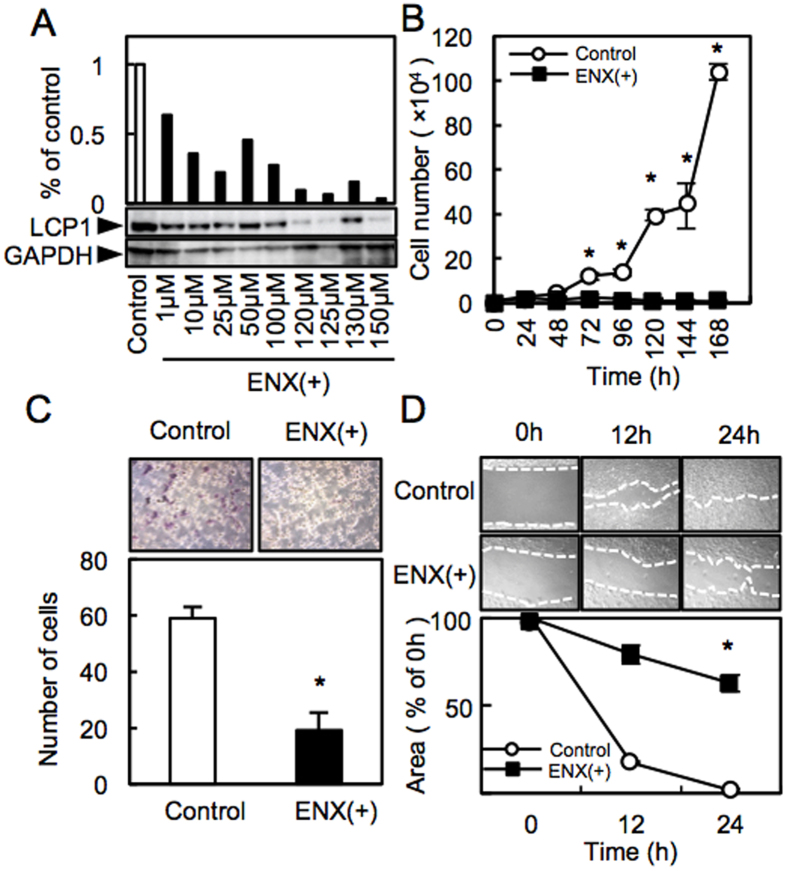
ENX treatment. **(A)** Immunoblot analysis of LCP1 protein levels in the ENX-treated cells. **(B)** Proliferation assay of the control and the ENX-treated cells. **(C)** Invasion assay of the control and the ENX-treated cells. **(D)** Migration assay of the control cells and the ENX-treated cells.

To investigate the efficiency of enoxacin (ENX), we assessed LCP1 expression and functional activities after treatment with ENX. Immunoblot analysis showed that LCP1 protein levels in the ENX-treated cells decreased obviously compared with the control cells (optimal concentration, 125 μM) (Fig. 4A). The cell growth of the ENX-treated cells was significantly inhibited compared with the control (Fig. 4B, *P* < 0.05, Student’s t-test). The number of ENX-treated cells invading the pores decreased significantly compared with the control (Fig. 4C, *P* < 0.05; Student’s t-test). In addition, the ENX-treated cells showed a wide gap after the 24 h treatment (Fig. 4D). These results indicated that ENX might regulate critical functions associated with tumoral growth and metastasis through down-regulation of LCP1.

### Correlation between LCP1 Expression and Clinical Classification in OSCCs

The correlations between the clinicopathologic features of OSCC cases and their LCP1 protein levels using the IHC scoring system were shown in Table 1. To determine the optimal cutoff value of the IHC scores, we performed receiver operating characteristic (ROC) curve analysis, which showed that the optimal cutoff value was 71.350 (area under the curve, 0.97; 95% confidence interval, 0.952–0.988). Among the clinical classifications, the LCP1 expression level showed a significant stratification in primary tumoral size (*P* = 0.016) and regional lymph node metastasis (*P* = 0.043).

## Discussion

We found that LCP1 was overexpressed in OSCC *in vitro* and *in vivo*; LCP1 knockdown cells decreased cell growth, invasiveness, and migratory activities; and LCP1 expression in clinical samples was associated positively with tumoral size and regional lymph node metastasis in OSCCs. Interestingly, we focused that an oral broad-spectrum fluoroquinolone, ENX, controlled LCP1 expression, leading to similar phenotypes of LCP1 knockdown cells.

Consistent with previous studies, the current study has shown the clinical relevance of LCP1 up-regulation, which is related closely to tumoral progression in various human cancers^1^,^3^,^4^,^5^,^6^,^7^,^8^. Normally, LCP1 expressed in leukocytes that responses to inflammatory and infections, therefore, LCP1 may be expressed in freely movable cells such as leukocytes and malignant cells^1^. In bladder cancer, tissue microarray analysis indicated that LCP1 expression was significantly correlated with tumor grade^11^. In addition to a previous study of prostate cancer progression^6^, our LCP1 knockdown models using OSCC cells is potentially useful to interfere with OSCC progression (Fig. 3). To date, the mechanism for the correlation between LCP1 and human malignancies is still unclear. In this regards, invasiveness for malignant melanoma and chemo-resistance for breast cancer may require phosphorylation of LCP1^9^,^12^. On the other hand, LCP-1 polymorphism (LCP1 rs494153) may be closely associated with its over-expression leading to predict colon cancer prognosis^5^. Thus, the mechanism for aberrant expression of LCP1 may differ from tumor to tumor, and more studies are needed to better understand the important role of LCP1 including its phosphorylation in tumoral progression.

Metastatic cancer cells use actin bundles to invade from the primary tumoral site through the surrounding tissue^4^. Immunofluorescence data showed that localization of F-actin, a binding partner of LCP1, was rearranged in shLCP1 cells (Fig. 2), which had low activity for cellular growth and tumoral invasion. Moreover, it is worthy to note that invasive carcinoma cells displayed strong immunoreaction for both LCP1 and F-actin (Supplementary Fig. S1). Therefore, our data suggested that the LCP1 together with F-actin, which may be formed LCP1-F-actin complex, has a role in proliferation and invasiveness of cancer cells.

ENX decreased cellular viability, induced apoptosis, caused cell cycle arrest, and inhibited the invasiveness in the prostate cancer cell lines^8^, making ENX an attractive candidate for use in cancer treatment as well as being an antibiotic. Comprehensive analysis using macrophages showed that LCP1 was down-regulated after treatment with ENX^13^, suggesting that ENX might regulate LCP1 expression. Our data indicated that ENX led to down-regulation of LCP1 and decreased cellular proliferation, invasiveness, and migratory activities. Further study is required to investigate if ENX is the upstream molecule of LCP1 in the cancer cells.

In conclusion, LCP1 can be a useful biomarker for determining the progression of OSCCs, and ENX might be a strong candidate as a new therapeutic agent against OSCCs by controlling LCP1 expression.

## Methods

### Ethics Statement

All experiments were performed in accordance with relevant guidelines and regulations. The ethics committee of Chiba University approved this study, protocol number, 236. We have obtained written informed consent from all subjects.

### OSCC-Derived Cell Lines and Tissue Specimens

Nine OSCC-derived cell lines, including HSC-2 (RBRC-RCB1945, mouth), HSC-3 (JCRB-0623, tongue), HSC-4 (RBRC-RCB1902, tongue), Sa3 (RBRC-RCB0980, upper gingiva), Ca9-22 (RCB-1976, gingiva), KOSC-2 (JCRB-0126.1, mouth floor), SAS (RBRC-RCB 1974, tongue), Ho-1-N-1 (JCRB-0831, buccal mucosa), and Ho-1-u-1 (RBRC-RCB2102, mouth floor), were purchased from the JCRB cell bank (Ibaraki, Osaka, Japan) and the RIKEN BioResource Center (Tsukuba, Ibaraki, Japan). We used, as described previously, primary cultured HNOKs as a normal control cells and tissue specimens^14^,^15^,^16^,^17^.

### mRNA Expression Analysis

We performed qRT-PCR as described previously^18^,^19^,^20^,^21^,^22^,^23^. Briefly, the primer sequences were: *LCP1*, forward, 5′*-*AAC CCT CGA GTC AAT CAT TTG*-*3′; reverse, 5′*-*TTT GAT CTT TTC ATA GAG CTG GAA*-*3′; probe, #37.

### Immunoblot Analysis

Immunoblot analysis was conducted as described previously^15^,^16^,^24^,^25^,^26^,^27^. The antibodies were affinity-purified mouse anti-LCP1 monoclonal antibody (sc-133219, Santa Cruz Biotechnology), rabbit anti-GAPDH monoclonal antibody (sc-25778, Santa Cruz Biotechnology), and mouse anti-F-actin monoclonal antibody (ab205, Abcam).

### IHC

IHC and IHC scoring systems were performed as described previously^10^,^21^,^22^,^26^,^27^,^28^,^29^,^30^,^31^. Briefly, the mean percentages of positive tumoral cells were determined in at least three random fields in each section, and the intensity of the LCP1 immunoreaction was scored using IHC profiler as follows: 0+, negative; 1+, low positive; 2+, positive; 3+, high positive. Moreover, we quantified the intensity of the LCP1 immunoreaction with IHC profiler, (https://souceforge.net/projects/ihcprofiler/)^32^. In order to determine the optimal cutoff point of LCP1 IHC scores, we evaluated the IHC scores from 121 samples with OSCC by constructing the ROC curve analysis for AUC calculation using LCP-1 expression in distinguishing oral cancer specimens from normal tissues. Cases with a score over the optimal cutoff point were defined as LCP1-positive^21^,^22^,^33^,^34^,^35^.

### Transfection with shRNA Plasmid

Transfection with shRNA Plasmid were conducted as described previously^16^,^21^,^22^. LCP1 shRNA (shLCP1) and control shRNA (shMock) vectors (sc-43209-SH, sc-108060, Santa Cruz Biotechnology) were transfected into Ca9-22 and Ho-1-N-1. After transfection, the cells were isolated and cultured as previously described^16^,^21^,^22^. To appraise the efficiency of LCP1 knockdown, we carried out qRT-PCR and immunoblotting.

### ENX Treatment

ENX, a fluoroquinolone, has been used extensively and with minimal side effects in humans to treat urinary tract infections and gonorrhea^36^. Several investigators reported that ENX down-regulated LCP1, resulting in decreased formation of actin rings. Therefore, we challenged the cells with ENX for functional analyses, such as cellular proliferation, invasiveness, and migration assays. Since Sousa *et al*. reported the half-maximal effective concentrations (105 and 141 μM) of ENX for two prostate cancer cell lines^8^, we performed immunoblotting using ENX (Tokyo Chemical) ranged from concentrations of 1 to 150 μM to determine the optimal concentration for further functional analyses.

### Functional Assay

Proliferation assay, invasion assay and migration assay was performed as described previously^14^,^17^,^21^,^22^,^37^,^38^,^39^,^40^.

### Immunofluorescence Analysis

IF was performed with a F-Actin Visualization Biochem Kit (Cytoskeleton) according to the manufacturer’s instructions and our protocol previously reported^22^,^40^,^41^. IF was observed using confocal microscopy and analyzed with the FluoView Software (Olympus Optical)^22^,^40^,^41^.

### Statistical Analysis

The statistical significance for LCP1 mRNA expression was calculated by the Student’s t-test. The correlations between the LCP1 IHC scores and each clinicopathological parameters were analyzed statistically by the χ^2^ test, Fisher’s exact test, and Mann-Whitney U-test. The significance level for two-sided P values was 0.05 for all tests. All data are expressed as the mean ± standard error of the mean of triplicate results.

## Additional Information

**How to cite this article**: Koide, N. *et al*. Evidence for Critical Role of Lymphocyte Cytosolic Protein 1 in Oral Cancer. *Sci. Rep.* **7**, 43379; doi: 10.1038/srep43379 (2017).

**Publisher's note:** Springer Nature remains neutral with regard to jurisdictional claims in published maps and institutional affiliations.

## Supplementary Material

Supplementary Information

## Figures and Tables

**Table 1:** Correlation between LCP1 expression and clinical classification in OSCCs.

Clinical classification	Total	Immunostaining results No. patients		*P* value
		LCP1-negative	LCP1-positive	
Age at surgery (years)				
<60	31	5	26	0.451^*^
≧60	90	12	78	
Gender				
Male	73	11	62	0.454^†^
Female	48	6	42	
T-primary tumor				
T1 + T2	46	11	35	0.016^†,‡^
T3 + T4	75	6	69	
N-regional lymph node				
Negative	66	13	53	0.043^†,‡^
Positive	55	4	51	
Vascular invasion				
Negative	83	12	71	0.849^†^
Positive	38	5	33	
Stage				
I + II	34	8	28	0.060^†^
III + IV	87	9	78	
Histopathologic type				
Well	80	14	66	0.126^§^
Moderately	33	2	31	
Poorly	8	1	7	
Tumoral site				
Tongue	63	9	54	0.546^§^
Gingiva	36	7	29	
Buccal mucosa	13	0	13	
Oral floor	7	1	6	
Lip	2	0	2	

*χ^2^ test.

^†^Fisher’s exact test.

^‡^*P* < 0.05.

^§^Mann-Whitney U-test.
