# Supplementary material for: Evidence for Critical Role of Lymphocyte Cytosolic Protein 1 in Oral Cancer
Source: Sci Rep. 2017 Feb 23;7:43379. doi: 10.1038/srep43379 (PMC5322526; doi:10.1038/srep43379)
Supplement: Supplementary Information [file srep43379-s1.pdf]

Supplementary Information

Evidence for Critical Role of Lymphocyte Cytosolic Protein 1 in Oral Cancer

Nao Koide[1], Atsushi Kasamatsu[2], Yosuke Endo-Sakamoto[2], Sho Ishida[1], Toshihiro Shimizu[3], Yasushi Kimura[4], Isao Miyamoto[5], Shusaku Yoshimura[1], Masashi Shiiba[6], Hideki Tanzawa[1,2], & Katsuhiro Uzawa[1,2]

[1]Department of Oral Science, Graduate School of Medicine, Chiba University, Chiba, Japan
[2]Department of Dentistry and Oral-Maxillofacial Surgery, Chiba University Hospital, Chiba, Japan
[3]Department of Oral Surgery, Kashima Rosai Hospital, Ibaraki, Japan
[4]Department of Oral and maxillofacial Surgery Research Institute, National Defense Medical College, Saitama, Japan
[5]Department of Dentistry and Oral-Maxillofacial Surgery, Japanese Red Cross Fukaya Hospital, Saitama, Japan
[6]Department of Clinical Oncology, Graduate School of Medicine, Chiba University, Chiba, Japan

*Corresponding Authors:
A. Kasamatsu and K. Uzawa, Department of Oral Science, Graduate School of Medicine, Chiba University, 1-8-1 Inohana, Chuo-ku, Chiba 260-8670, Japan
E-mails: kasamatsua@faculty.chiba-u.jp; uzawak@faculty.chiba-u.jp

Supplemental Fig. S1

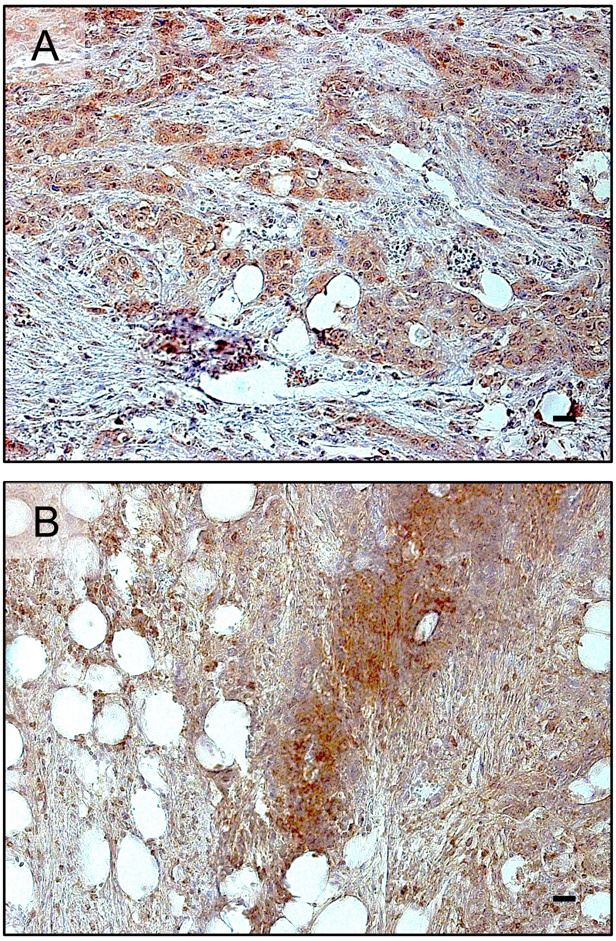

Supplemental Figure S1. Representative results of IHC for LCP1 (A) and F-actin (B). Note that the cancer cells at the invasive front reveal strong immunoreaction for both LCP1 and F-actin. Original magnification, x 200. Scale bars, 50 μm.
